# Internalization Dissociates β_2_-Adrenergic Receptors

**DOI:** 10.1371/journal.pone.0017361

**Published:** 2011-02-22

**Authors:** Tien-Hung Lan, Sudhakiranmayi Kuravi, Nevin A. Lambert

**Affiliations:** Department of Pharmacology and Toxicology, Georgia Health Sciences University, Augusta, Georgia, United States of America; University of Hong Kong, Hong Kong

## Abstract

G protein-coupled receptors (GPCRs) self-associate as dimers or higher-order oligomers in living cells. The stability of associated GPCRs has not been extensively studied, but it is generally thought that these receptors move between the plasma membrane and intracellular compartments as intact dimers or oligomers. Here we show that β_2_-adrenergic receptors (β_2_ARs) that self-associate at the plasma membrane can dissociate during agonist-induced internalization. We use bioluminescence-resonance energy transfer (BRET) to monitor movement of β_2_ARs between subcellular compartments. BRET between β_2_ARs and plasma membrane markers decreases in response to agonist activation, while at the same time BRET between β_2_ARs and endosome markers increases. Energy transfer between β_2_ARs is decreased in a similar manner if either the donor- or acceptor-labeled receptor is mutated to impair agonist binding and internalization. These changes take place over the course of 30 minutes, persist after agonist is removed, and are sensitive to several inhibitors of arrestin- and clathrin-mediated endocytosis. The magnitude of the decrease in BRET between donor- and acceptor-labeled β_2_ARs suggests that at least half of the receptors that contribute to the BRET signal are physically segregated by internalization. These results are consistent with the possibility that β_2_ARs associate transiently with each other in the plasma membrane, or that β_2_AR dimers or oligomers are actively disrupted during internalization.

## Introduction

Abundant evidence suggests that G protein-coupled receptors (GPCRs) can self-associate as dimers or higher-order oligomers. Although signaling to heterotrimeric G proteins and arrestins does not require self-association [1], [2], [3], several functional interactions between receptors have been attributed to oligomerization. Among the processes thought to be influenced by oligomerization are trafficking of GPCRs to the plasma membrane during biosynthesis, and internalization following ligand activation [4]. Several studies have shown that receptors that fail to reach the plasma membrane during biosynthesis can exert a dominant negative effect that leads to intracellular retention of other receptors [4], [5]. In other cases forward trafficking of poorly-expressed receptors is facilitated by expression of other GPCRs [6]. Similarly, in several instances agonist-induced internalization of a given GPCR has been shown to promote internalization of either a disabled mutant or a different GPCR altogether [7], [8], [9], [10], [11]. These observations have led to the general conclusion that GPCRs traffic to and from the plasma membrane as intact homo- and heterooligomers.

This conclusion is consistent with the concept that GPCRs associate with each other as thermodynamically stable dimers and higher-order oligomers. However, it has recently been suggested that several GPCRs self-associate transiently on the cell surface [12], [13], [14], raising the possibility that such GPCRs might not traffic as intact dimers or oligomers. This possibility prompted us to investigate the self-association of β_2_-adrenergic receptors (β_2_ARs) during agonist-induced internalization, as these receptors are prototypical for both oligomerization [15] and agonist-induced internalization [16]. Here we show that agonist-induced internalization segregates active and inactive β_2_ARs that are initially in close proximity at the plasma membrane. This result is consistent with the possibility that β_2_AR dimers or oligomers are not completely stable on the cell surface.

## Results

### BRET reports β_2_AR internalization

**Figure 1:**
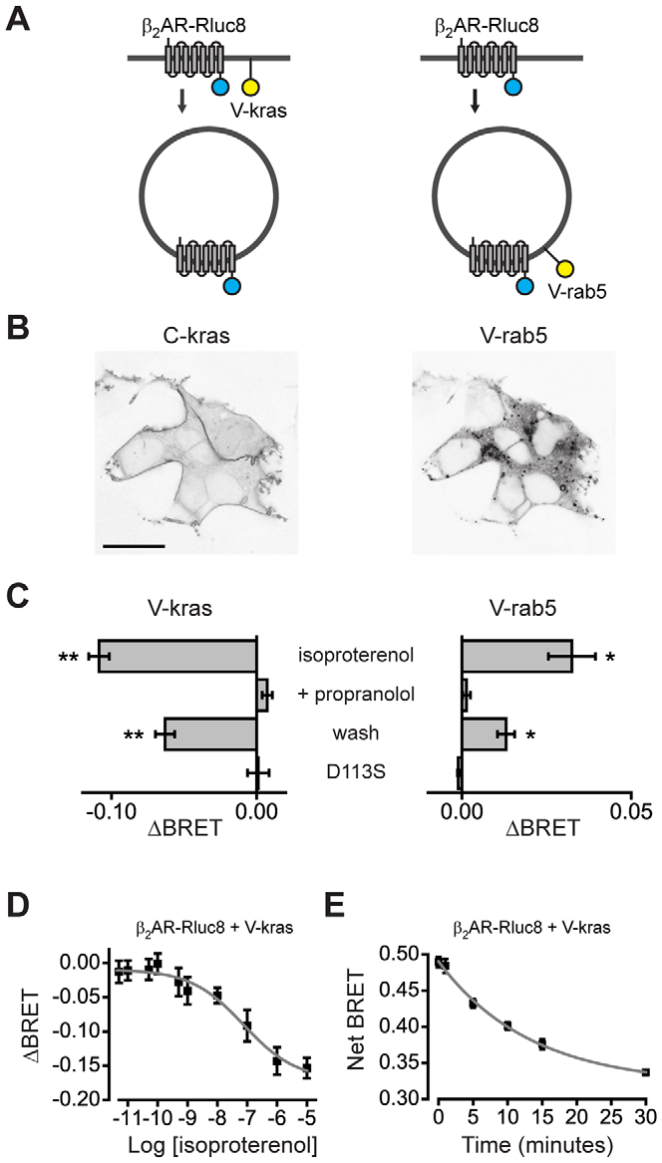
BRET reports β_2_AR internalization. **A**, schematic representation of the location and orientation of β_2_AR-Rluc8, the plasma membrane acceptor venus-kras (V-kras), and the early endosome acceptor venus-rab5 (V-rab5). **B**, confocal images of cells expressing cerulean-kras (C-kras) and V-rab5; the kras and rab5 localization signals direct fluorescent proteins to distinct compartments consistent with the plasma membrane and early endosomes, respectively. Scale bar  = 20 µm. **C**, changes in BRET (ΔBRET) between β_2_AR-Rluc8 and V-kras (*left*) and V-rab5 (*right*) after 30 minute incubation with 10 µM isoproterenol, isoproterenol plus 10 µM propranolol, 30 minutes after isoproterenol removal (wash), or after isoproterenol with β_2_AR D113S-Rluc8 (*n* = 4 experiments performed in quadruplicate); *, *P*<0.05; **, *P*<0.005, paired t-test, meaning the isoproterenol-induced ΔBRET is significantly different from zero. Values of net BRET are given in Table S1. **D**, changes in BRET between β_2_AR-Rluc8 and V-kras as a function of isoproterenol concentration. The smooth line is a least-squares fit to a Hill equation (EC_50_ = 75 nM; *n* = 4). **E**, net BRET between β_2_AR-Rluc8 and V-kras as a function of time after addition of isoproterenol; BRET was measured after isoproterenol was removed and replaced with 10 µ µM propranolol. The smooth line is a least-squares fit to a single exponent (τ = 13.3 minutes; *n* = 4).

In order to study β_2_AR self-association and internalization together in intact cells we used an assay in which resonance energy transfer between receptors and an inert plasma membrane marker is measured with and without agonist application [17]. In this application BRET is used not to indicate a protein-protein interaction, but rather as an indicator of subcellular location. The *Renilla* luciferase Rluc8 was fused to the C-terminus of the β_2_AR (β_2_AR-Rluc8) to serve as a BRET donor. The fluorescent protein venus was fused to the N-terminus of a C-terminal fragment of KRas (V-kras) to serve as a plasma membrane-associated BRET acceptor. Transient expression of these two proteins in HEK 293 cells produced a substantial BRET signal, as expected for two proteins that are associated with the plasma membrane. Activation of β_2_AR-Rluc8 by pretreating with isoproterenol (10 µM) for 30 minutes induced a significant decrease in BRET compared to vehicle-treated controls, consistent with the removal of a fraction of these receptors from the plasma membrane (Figure 1A, C) [16]. Net BRET between β_2_AR-Rluc8 and V-kras was 0.26±0.02 in control cells and 0.15±0.02 in isoproterenol-treated cells (ΔBRET  =  -0.11±0.01, *n* = 5, performed in quadruplicate; Table S1). BRET measurements were made immediately after cells were washed and harvested in agonist-free buffer, thus the decrease in BRET did not depend on continued activation of cell surface β_2_AR-Rluc8. This decrease was prevented if the antagonist propranolol (10 µM) was applied at the same time as isoproterenol, and was partially reversed if isoproterenol was removed for 30 minutes prior to harvesting cells and measuring BRET (Figure 1C). In addition, no significant isoproterenol-induced BRET change was observed when β_2_AR-Rluc was mutated (D113S) [18] to prevent high-affinity isoproterenol binding (Figure 1C). The isoproterenol-induced BRET decrease was concentration-dependent, with an EC_50_ of 75 nM (Figure 1D).

**Figure 2:**
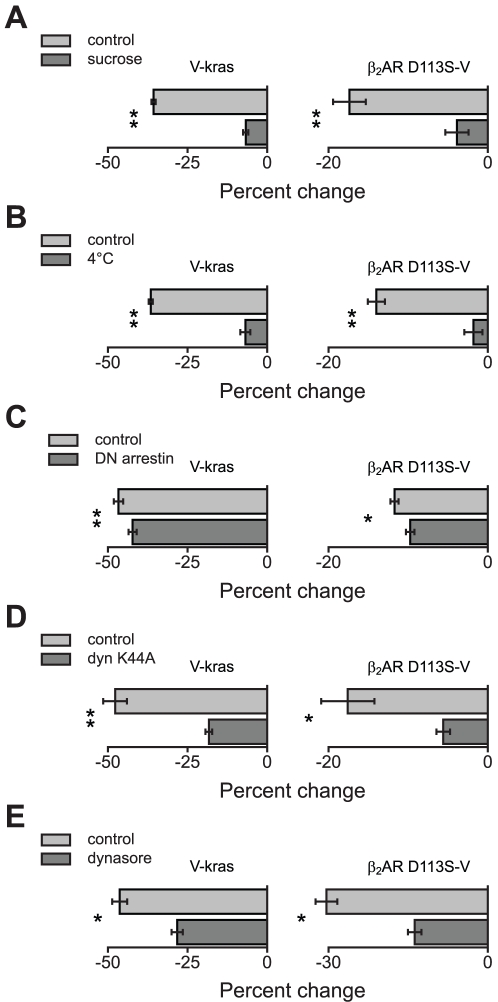
Isoproterenol-induced changes in BRET are sensitive to inhibitors of clathrin-mediated endocytosis. **A–E**, the percent change in net BRET induced by isoproterenol is plotted for cells expressing β_2_AR-Rluc8 and V-kras (*left*) or β_2_AR-Rluc8 and β_2_AR D113S-V (*right*). Cells were pretreated with vehicle or were cotransfected with vector (control), or were pretreated with 400 mM sucrose (**A**; *n* = 5 and 5), incubated at 4°C (**B**; *n* = 5 and 6), cotransfected with arrestin2 (319–418) (**C**; *n* = 6 and 6), cotransfected with dynamin K44A (**D**; *n* = 5 and 5), or pretreated with dynasore (80 µM; **E**; *n* = 4 and 4); **, *P*<0.005, paired t-test, meaning the percent change in net BRET in the presence of sucrose, etc., is significantly different from the control percent change in net BRET. Values of net BRET are given in Table S2 and Table S4.

Several observations suggested that the isoproterenol-induced decrease in BRET between β_2_AR-Rluc8 and V-kras was due to arrestin- and dynamin-dependent internalization of β_2_AR-Rluc8. For example, we found that isoproterenol produced a reciprocal increase in BRET between β_2_AR-Rluc8 and venus-labeled rab5a (V-rab5; Figure 1A, C), which is associated with early endosomes (Figure 1B) and clathrin-coated pits [19]. In addition, the isoproterenol-induced BRET decrease developed over the course of 30 minutes, and persisted when intact cells were washed and then exposed to the antagonist propranolol (Figure 1E). These features are consistent with previous studies of β_2_AR internalization [16], [17], and are not consistent with the change in BRET ratio simply being due to a change in relative distance/orientation of the donor and acceptor on the cell surface. To test this conclusion further we employed several methods that are known to inhibit clathrin-mediated endocytosis. The isoproterenol-induced decrease in BRET between β_2_AR-Rluc8 and V-kras was significantly inhibited by pretreatment with hypertonic sucrose (Figure 2A), preincubation of cells at 4°C (Figure 2B), coexpression of dominant-negative arrestin2 (319-418) [20] (Figure 2C), coexpression of dominant-negative dynamin K44A [21] (Figure 2D), and pretreatment with the small molecule dynamin inhibitor dynasore (80 µM) [22] (Figure 2E). In terms of efficacy, sucrose and 4°C incubation were the most effective inhibitors of the BRET decrease, arrestin2 (319–418) and dynasore were the least effective, and dynamin K44A was intermediate. In some cases (e.g. with expression of arrestin2 (319–418)) these treatments changed net BRET in control cells not exposed to isoproterenol (Table S2). These results indicate that BRET, like FRET [17], is a sensitive reporter of agonist-induced β_2_AR internalization in living cells.

### Interactions between active and inactive β_2_ARs during internalization

**Figure 3:**
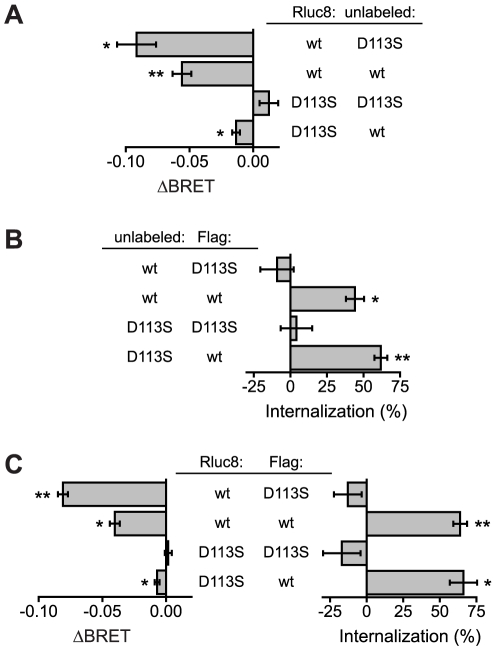
Interactions between active and inactive (binding-defective) β_2_ARs during internalization. **A**, decreases in BRET (ΔBRET) after isoproterenol treatment in cells expressing two different β_2_ARs. Either wild-type (wt) or binding-defective (D113S) β_2_AR-Rluc8 were expressed with either wt or D113S unlabeled β_2_AR and V-kras (*n* = 4). Modest but significant internalization of β_2_AR D113S-Rluc8 is promoted by unlabeled wt β_2_AR. **B**, loss of cell surface Flag immunoreactivity in cells expressing either wild-type (wt) or binding-defective (D113S) Flag-β_2_AR together with either wt or D113S unlabeled β_2_AR (*n* = 4). No detectable internalization of Flag-β_2_AR D113S was promoted by unlabeled wt β_2_AR. **C**, decreases in BRET (*left*) and loss of cell surface Flag immunoreactivity (*right*) after isoproterenol treatment in cells expressing the indicated combinations of β_2_AR-Rluc8 and Flag-β_2_AR (*n* = 4–5). *, *P*<0.05; **, *P*<0.005, paired t-test, meaning ΔBRET or % internalization is significantly different from zero. Values of net BRET are given in Table S3.

We next used BRET to look for interactions between β_2_ARs during internalization. Specfically, we wanted to know if inactive β_2_ARs could impede internalization of active β_2_ARs, or alternatively if active β_2_ARs could promote internalization of inactive β_2_ARs [7], [8], [10], [11]. Wild-type (wt) β_2_AR-Rluc8 was coexpressed with V-kras and either the unlabeled binding-defective mutant β_2_AR D113S [18] or unlabeled wild-type β_2_AR. In both cases a five-fold excess of plasmid DNA expressing the unlabeled receptor was transfected in order to maximize the likelihood that Rluc8-labeled receptors would associate with unlabeled receptors. The presence of binding-defective β_2_AR D113S did not impede internalization of β_2_AR-Rluc8 as determined by the isoproterenol-induced decrease in BRET (Figure 3A). In fact, internalization was significantly greater with coexpression of β_2_AR D113S than with coexpression of β_2_AR (*P*<0.05, repeated measures ANOVA). The underlying cause of this difference is not clear, but it may reflect competition between β_2_AR-Rluc8 and β_2_AR for limited internalization capacity. Alternatively, this difference may reflect negative allosteric interactions between active receptor protomers, as has been described for other GPCRs [23]. Not surprisingly, no isoproterenol-induced internalization was detected when both Rluc8-labeled and unlabeled β_2_ARs were binding-defective D113S mutants. In contrast, modest but significant internalization of β_2_AR D113S-Rluc8 was detected when wt β_2_AR was present (Figure 3A). When expressed as a percentage of the control net BRET between β_2_AR-Rluc8 and V-kras, 44%±3% of the signal was lost after internalization if β_2_AR-Rluc8 was active and unlabeled β_2_AR was inactive, whereas only 6%±1% of the signal was lost if β_2_AR-Rluc8 was inactive and unlabeled β_2_AR was active. This result is consistent with previous reports where wild-type GPCRs promoted the agonist-induced internalization of binding- or internalization-defective receptors [7], [8], [9], [10], [11]. However, our quantitative results suggest that the efficiency of this process is low.

The weak influence of wild-type β_2_AR on internalization of β_2_AR D113S-Rluc8 prompted us repeat the same basic experiment using a more conventional method of detecting internalization. Accordingly, Rluc8-labeled receptors were replaced with N-terminally Flag-tagged receptors (Flag-β_2_AR), and internalization was measured by assaying loss of cell-surface Flag immunoreactivity by ELISA. The results of this experiment agreed well with our BRET results (Figure 3B), with the exception that this assay failed to detect significant agonist-induced internalization of Flag-β_2_AR D113S in the presence of β_2_AR.

Finally, we combined the two detection methods by expressing Rluc8-labeled receptors and Flag-tagged receptors together (with V-kras). This combination allowed us to verify that wild-type β_2_ARs internalized in response to agonist while at the same time binding-defective β_2_ARs largely remained on the cell surface using two independent methods. Accordingly, BRET signals decreased significantly in response to isoproterenol when Rluc8-labeled receptors were wild-type, but far less or not at all if Rluc8-labeled receptors were binding-defective (Figure 3C). Cell surface Flag immunoreactivity decreased significantly only when Flag-tagged receptors were binding-competent (Figure 3C).

### Internalization segregates active and inactive β_2_ARs

**Figure 4:**
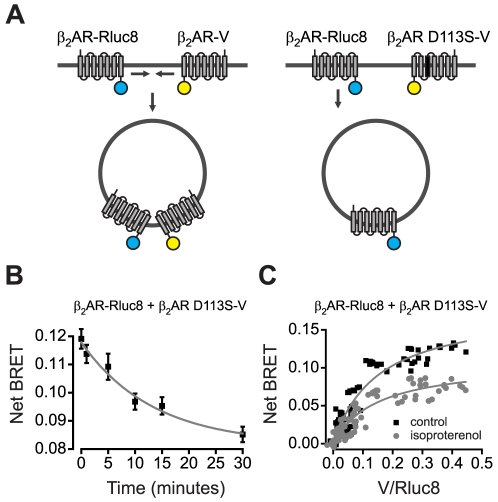
Internalization segregates active and inactive β_2_ARs. **A**, schematic representation of recruitment of wild-type (wt) β_2_AR-Rluc8 and wt β_2_AR-V to coated pits and internalization (*left*), and segregation of wt β_2_AR-Rluc8 and β_2_AR D113S-V (*right*). **B**, net BRET between wt β_2_AR-Rluc8 and β_2_AR D113S-V as a function of time after addition of isoproterenol; BRET was measured after isoproterenol was removed and replaced with 10 µM propranolol. The smooth line is a least-squares fit to a single exponent (τ = 13.3 minutes; *n* = 4). **C**, net BRET between wt β_2_AR-Rluc8 and β_2_AR D113S-V with increasing expression of the acceptor in control and isoproterenol-treated cells. Smooth curves are least-squares fits to a hyperbolic function; the derived V/Rluc8 intensity ratio at which net BRET was half-maximal (BRET_50_) was 0.15 in both cases, thus the agonist-induced decrease BRET decrease did not depend on V/Rluc8.

In light of the evidence that β_2_ARs self-associate in the plasma membrane, our results indicating only modest functional interactions between wild-type and binding-defective β_2_ARs during internalization suggested that some active and inactive receptors segregate during this process. However, in these experiments it was not possible to directly verify the expectation that wild-type and binding-defective β_2_ARs associated with each other in dimers or higher order oligomers. Therefore, to test the idea of protomer segregation more directly we measured changes in BRET between β_2_AR-Rluc8 and the venus-labeled acceptor β_2_AR-V (and binding defective mutants of these receptors) due to stimulation with isoproterenol (Figure 4A).

Substantial BRET was observed when β_2_AR-Rluc8 and β_2_AR-V were coexpressed, as has been reported previously [24]. Basal net BRET (without isoproterenol treatment) between wild-type β_2_AR-Rluc8 and wild-type β_2_AR-V (0.20±0.02; *n* = 27) and that between wild-type β_2_AR-Rluc8 and β_2_AR D113S-V (0.18±0.01; *n* = 26; *P* = 0.19, unpaired t-test) were not significantly different, suggesting that the D113S mutation did not interfere with β_2_AR maturation or association [11], [18]. When the donor was wild-type β_2_AR-Rluc8 and the acceptor was binding-defective β_2_AR D113S-V, isoproterenol reliably induced a significant BRET decrease (to 0.15±0.01; *P*<0.001, paired t-test). As was the case with the plasma membrane-associated acceptor V-kras, the agonist-induced decrease in BRET between β_2_AR-Rluc8 and β_2_AR D113S-V developed over the course of 30 minutes (Figure 4B; cf. Figure 1E), and persisted when cells were washed with and exposed to the antagonist propranolol. As was the case with the V-kras, all five methods of inhibiting clathrin-mediated endocytosis significantly inhibited the agonist-induced decrease in BRET to β_2_AR D113S-V, and in each case the degree of inhibition was similar to what was observed when the donor was V-kras (Figure 2A–E; Table S4). Susceptibility to these manipulations indicates that this agonist-induced BRET change is largely due to arrestin- and dynamin-dependent processes, as opposed to conformational changes within or between associated β_2_ARs. Similar results were obtained when the donor was binding-defective β_2_AR D113S-Rluc8 and the acceptor was wild-type β_2_AR-V (DS/wt in Figure S1), but not when both donor and acceptor were binding defective (β_2_AR D113S-Rluc8 and β_2_AR D113S-V; DS/DS in Figure S1). Taken together these results are consistent with a model wherein active wild-type β_2_AR internalizes in response to agonist, while inactive β_2_AR D113S remains in the plasma membrane (Figure 4A).

Interestingly, when both β_2_AR-Rluc8 and β_2_AR-V were wild-type isoproterenol reliably induced a significant increase in BRET (to 0.22±0.02; *P*<0.0001, paired t-test; wt/wt in Figure S1), as reported previously [24]. One possible explanation for this is that both donor- and acceptor-labeled receptors were concentrated in clathrin-coated pits and vesicles, as the BRET increase was significantly reduced by manipulations known to inhibit clathrin assembly and by dynamin K44A (Figure S1). Surprisingly, the dynamin inhibitor dynasore enhanced the agonist-induced BRET increase between wild-type β_2_ARs. The reason for the difference between the effects of dynamin K44A and dynasore on agonist-induced BRET between wild-type β_2_ARs is not obvious. However, these two agents are known to act at different stages of clathrin-coated pit formation [22], [25], thus it is possible that dynasore traps receptors in newly-formed coated pits, whereas dynamin K44A prevents recruitment to coated pits altogether.

BRET signals between β_2_AR-Rluc8 and β_2_AR-V could arise from specific association of individual protomers to form dimers, specific association of dimers to form higher-order oligomers, or non-specific colocalization of any of these elements. Internalization of active receptors could conceivably disrupt any or all of these modes of association. We considered the possibility that internalization disrupted only non-specific colocalization of β_2_ARs by measuring BRET between β_2_AR-Rluc8 and β_2_AR D113S-V across a range of acceptor/donor ratios, as removal of a non-specific component would be predicted to lead to saturation of BRET at a lower acceptor/donor ratio [26]. As shown previously [27], net BRET increased hyperbolically as the relative expression of β_2_AR D113S-V increased for both control and isoproterenol-treated cells (Figure 4C). The acceptor/donor ratio at which BRET was half-maximum (BRET_50_) was 0.15 arbitrary units for both groups of cells, thus the isoproterenol-induced BRET decrease did not depend on the acceptor/donor ratio. This result suggests that the agonist-induced decrease in BRET between β_2_AR-Rluc8 and β_2_AR D113S-V was not due to the selective elimination of a non-specific signal, and that the average affinity of β_2_AR association was the same in agonist-treated and control cells.

## Discussion

The results of this study suggest that the association of β_2_ARs is not permanent in living cells, and that active and inactive β_2_ARs that are associated at the cell surface dissociate during agonist-induced internalization of active protomers.

Most previous studies have concluded that GPCRs internalize in response to agonist as intact dimers, even when only one protomer binds ligand [7], [8], [10], [11]. For example, studies in the yeast *Saccharomyces cervisiae* have reported that internalization of wild-type Ste2 receptors promoted internalization of internalization- or binding-defective receptors [7], although this process was less efficient than expected for completely stable dimers [10]. Similarly, internalization of opioid receptors can promote internalization of other opioid receptors or β_2_ARs, and internalization of β_2_ARs can promote internalization of opioid receptors [28], [29]. These studies support the general conclusion that GPCRs internalize as intact hetero- and homodimers or oligomers after activation of a single protomer [4]. However, in other cases activation of one protomer either fails to induce internalization of heterodimers (e.g. δ-κ opioid heterodimers) [30], or promotes dissociation of heterodimers (e.g. endothelin A-endothelin B heterodimers) [31].

In the present study we provide direct evidence that a substantial fraction of the interactions that contribute to energy transfer between active and inactive β_2_ARs in intact cells is disrupted by internalization. Our results appear to be at odds with those of Sartania et al. [11], who concluded that β_2_ARs internalize as intact homodimers. However, this study did not quantitate the fraction of β_2_ARs that internalize as dimers, and did not rule out disruption of a fraction of β_2_AR assemblies. Our results are also somewhat surprising in light of reports which have shown that β_2_ARs with altered trafficking itineraries can efficiently impede the cell surface expression and recycling of wild-type β_2_ARs [5], [32]. Taken together these studies suggest that the interaction between receptor trafficking and oligomerization may differ for the various receptors and for various stages of the receptor life cycle.

What fraction of interactions between β_2_ARs is disrupted by internalization? Previous studies using methods similar to those used here have shown that, at steady-state, agonist-induced internalization decreases the number of β_2_ARs in the plasma membrane by about one-half to two-thirds [16], [33]. We observed a similar decrease in the present study using a cell-surface ELISA assay (Figure 3). Therefore, the upper limit to the decrease in net BRET one could expect due to internalization is within this range. In our experiments 25–30% of the net BRET signal between β_2_AR-Rluc8 and β_2_AR D113S-V was lost after stimulation with isoproterenol, thus we conclude that at least half of the associations that contribute to BRET at the cell surface can be disrupted by internalization. This is almost certainly an underestimate, since a fraction of the BRET signal that we measure prior to agonist stimulation presumably originates from β_2_ARs in intracellular compartments, and would not be expected to change in response to agonist stimulation. For example, if half of the total BRET signal originated from intracellular compartments prior to agonist stimulation, then only 25% of the total BRET signal could be lost due to internalization of 50% of the receptors originally present on the plasma membrane. Methods capable of quantifying association of active and inactive β_2_ARs specifically in the plasma membrane will be necessary to make a more precise estimate of the fraction of interactions that are disrupted by internalization [34].

What is the nature of the interactions that are disrupted by internalization? When expression of β_2_AR D113S-V was increased (with constant expression of β_2_AR-Rluc8) the net BRET signal approached a maximum [27]. The standard interpretation of this finding is that random association favors donor:donor dimers at low acceptor concentrations, and donor:acceptor dimers at high acceptor concentration, and a maximum is reached when all donors are associated with acceptors. This interpretation relies on the assumption that the transmembrane domains of the β_2_AR determine dimer assembly irrespective of binding site mutations or fused reporter proteins. If this is the case, then our results imply that internalization disrupts some β_2_AR dimers where one protomer is active (β_2_AR-Rluc8) and the other protomer is inactive (β_2_AR D113S-V). We cannot rule out the possibility that some donor:donor dimers are present even when the acceptor concentration is very high. In this case some (or all) of the internalization-sensitive BRET could reflect removal of β_2_AR-Rluc8: β_2_AR-Rluc8 dimers from higher-order oligomers that contain β_2_AR D113S-V protomers in close proximity. However, the fact that BRET_50_ was not changed after agonist-induced internalization implies that the average stability of β_2_AR associations is the same before and after internalization. The simplest interpretation of this finding is that internalization disrupts a fraction of a homogeneous class of interactions, although more complex scenarios are certainly possible. In either case, our results are difficult to reconcile with a model wherein β_2_ARs randomly form dimers that remain stable during internalization driven by activation of a single protomer.

The simplest model that accomodates our findings would be that β_2_ARs associate with each other transiently, such that there is a monomer-dimer equilibrium on the cell surface. Recruitment of active protomers to clathrin-coated pits would lead to passive secondary recruitment of inactive protomers. However, if β_2_AR protomers dissociate at a rate comparable to the rate of recruitment to coated pits, the ratio of active (wild-type) to inactive (e.g. binding-defective) protomers would be higher in coated pits and vesicles than on the unstimulated cell surface. This would lead to selective internalization of active protomers, the degree of selectivity being determined by the stability of the protomer-protomer interaction. This mechanism would be consistent with the suggestion that some GPCRs associate with each other only transiently on the cell surface [12], [13], [14]. We can not exclude a more complicated scenario that involves a more active mechanism of protomer segregation. Such an active mechanism would be necessary if, as has been suggested, β_2_ARs form relatively stable oligomers [12].

In summary, we find that the interactions between β_2_ARs that produce energy transfer are not entirely stable. Agonist stimulation leads to internalization of active β_2_AR protomers, while inactive protomers remain in the plasma membrane, even if these protomers were initially in close proximity to one another. These findings suggest that the impact of GPCR self-association on the trafficking of individual protomers or dimers may differ depending on the particular receptor and trafficking step involved.

## Materials and Methods

### Plasmid DNA constructs

A plasmid encoding Rluc8 [35] was provided by Dr. Sanjiv Sam Gambhir (Stanford University, Palo Alto, CA). A plasmid encoding venus-kras was provided by Dr. Stephen R. Ikeda (NIAAA, Rockville, MD). A plasmid encoding arrestin2 (319–418) was provided by Dr. Jeffrey L. Benovic (Thomas Jefferson University, Philadelphia, PA). Fusion proteins were constructed and mutations were introduced using a variation of the QuikChange (Stratagene, La Jolla, CA) mutagenesis protocol. All constructs were verified by automated sequencing.

### Cell culture, transfection, and ligand treatment

HEK 293 cells (ATCC, Manassas, VA) were propagated in plastic flasks, in 6-well plates and on polylysine-coated glass coverslips according to the supplier's protocol. Cells were transfected in growth medium using linear polyethyleneimine (MW 25,000; Polysciences Inc., Warrington, PA) at an N/P ratio of 20; up to 3 µg of plasmid DNA was transfected per well of a 6-well plate. Cells were used for experiments 16–24 hours after transfection. Isoproterenol was applied to adherent cells in complete growth medium and incubated at 37°C (unless otherwise indicated) and 5% CO_2_ for 30 minutes. As indicated, prior to addition of isoproterenol some cells were: supplemented with 50 mM NaHEPES (pH 7) and maintained at 4°C for 15 minutes; supplemented with 400 mM sucrose for 15 minutes; treated with either dynasore (80 µM) or vehicle (DMSO) for 30 minutes.

### BRET measurements

Cells were detached from plates by rinsing with PBS-EDTA and triturating in PBS. For time course experiments (Figures 1E and 3B) cells were washed and suspended in PBS containing 10 µM propranolol. Suspended cells were transferred to black 96-well microplates. Coelenterazine h (5 µM; Nanolight Technologies, Pinetop, AZ) was added to all wells immediately prior to making measurements. Luminescence measurements were made using a photon-counting plate reader (Mithras LB940; Berthold Technologies GmbH, Bad Wildbad, Germany). The raw BRET signal was calculated as the emission intensity at 520–545 nm divided by the emission intensity at 475–495 nm. Net BRET was this ratio minus the same ratio measured from cells expressing only the BRET donor (Rluc8). Fluorescence intensity was measured prior to addition of coelenterazine h. Acceptor fluorescence and donor luminescence were monitored to ensure comparable levels of β_2_AR expression unless expression was intentionally varied (Figure 3C).

### Confocal imaging

Confocal images (Figure 1) were acquired using a Leica (Wetzlar, Germany) SP2 scanning confocal microscope and a 63X, 1.4 NA objective. Cerulean and venus were excited with the 458 nm and 514 nm lines of an ArKr laser, and detected at 465–490 nm 520–550 nm, respectively.

### Quantification of cell-surface β_2_ARs by ELISA

Cells were washed with PBS, then blocked in ice-cold PBA (1% bovine serum albumin, 0.1% sodium azide in PBS) for 30 minutes. Cells were incubated in 1∶1000 M2 anti-FLAG monoclonal antibody (Stratagene, La Jolla, CA) in PBA for 30 minutes at room temperature, washed three times with PBS, then incubated in 1∶1000 HRP-conjugated goat anti-mouse secondary antibody (Jackson ImmunoResearch, West Grove, PA) in PBA for 30 minutes at room temperature. After washing in PBS cells were detached from plates and distributed into white 96-well plates. Chemiluminescent substrate (Pierce SuperSignal ELISA Pico, Thermo Fisher Scientific, Rockford, IL) was added according to the manufacturer's instructions. Readings were background-subtracted using samples exposed only to the secondary antibody. The average signal/background ratio for control cells was 54±10 (*n* = 16). Signals were normalized to cell number, which was estimated from measurements of FM1-43 fluorescence.

### Statistical analysis

Comparisons involving two sets of data were made using either a paired or unpaired student's t-test. Comparisons involving more than two sets of data were made using a repeated measures ANOVA. Each transient transfection was treated as an individual subject, and pairing by subjects was significant for both paired t-tests and repeated measures ANOVA. All data are reported in the text and figures as mean ± S.E.M. All experiments were performed in quadruplicate.

## Supporting Information

Figure S1 **Internalization segregates active and inactive β_2_ARs.** **A–E**, isoproterenol-induced percent change in net BRET between wt β_2_AR-Rluc8 and wt β_2_AR-V (wt/wt), wt β_2_AR-Rluc8 and β_2_AR D113S-V (wt/DS), β_2_AR D113S-Rluc8 and wt β_2_AR-V (DS/wt) and β_2_AR D113S-Rluc8 and β_2_AR D113S-V (DS/DS) in the absence (control) and presence of 400 mM sucrose (**A**; *n* = 5–6), incubation at 4°C (**B**; *n* = 3–6), coexpressed arrestin2 (319–418) (**C**; *n* = 6–7), coexpressed dynamin K44A (**D**; *n* = 5), dynasore (80 µM; **E**; *n* = 3–4); *, *P*<0.05; **, *P*<0.005, paired t-test, meaning the percent change in net BRET in the presence of sucrose, etc., is significantly different from the control percent change in net BRET. Values of net BRET are given in Table S4.
(EPS)

Table S1 **net BRET between β_2_AR-Rluc8 or β_2_AR D113S-Rluc8 and V-kras (** **Figure 1C** **).**
(DOC)

Table S2 **net BRET between β_2_AR-Rluc8 and V-kras (** **Figure 4** **).**
(DOC)

Table S3 **net BRET between β_2_AR-Rluc8 wild-type (wt) and binding-defective (DS) mutants and V-kras (** **Figure 2A, C** **).**
(DOC)

Table S4 **net BRET between β_2_AR-Rluc8 and β2AR-V wild-type (wt) and binding-defective (DS) mutants (** **Figure 4** **and Figure S1).**
(DOC)
