# Supplementary material for: Internalization Dissociates β2-Adrenergic Receptors
Source: PLoS One. 2011 Feb 22;6(2):e17361. doi: 10.1371/journal.pone.0017361 (PMC3043075; doi:10.1371/journal.pone.0017361)
Supplement: Table S1 — net BRET between β2AR-Rluc8 or β2AR D113S-Rluc8 and V-kras ( Figure 1C ). (DOC) [file pone.0017361.s002.doc]

Table S1: net BRET between β_2_AR-Rluc8 or β_2_AR D113S-Rluc8 and V-kras (Figure 1C).

|  |  | **V-kras** | | | | **V-rab5** | | | |
| --- | --- | --- | --- | --- | --- | --- | --- | --- | --- |
| **donor:** | ***n*** | **control** | **isoproterenol** | **+ propranolol** | **wash** | **control** | **isoproterenol** | **propranolol** | **wash** |
| **β2AR-Rluc8** | 5 | 0.259 ± 0.023 | 0.151 ± 0.017† | 0.267 ± 0.024‡ | 0.196 ± 0.020†‡ | 0.034 ± 0.003 | 0.066 ± 0.010† | 0.035 ± 0.004‡ | 0.047 ± 0.006‡ |
| **β2AR D113S-Rluc8** | 5 | 0.324 ± 0.019 | 0.326 ± 0.022 | 0.335 ± 0.025 | 0.323 ± 0.028 | 0.045 ± 0.004 | 0.043 ± 0.004 | 0.047 ± 0.003 | 0.047 ± 0.003‡ |

†- significantly different from control; *P*<0.05, repeated measures ANOVA followed by Tukey’s multiple comparison.

‡- significantly different from isoproterenol; *P*<0.05, repeated measures ANOVA followed by Tukey’s multiple comparison.
