# Supplementary material for: Internalization Dissociates β2-Adrenergic Receptors
Source: PLoS One. 2011 Feb 22;6(2):e17361. doi: 10.1371/journal.pone.0017361 (PMC3043075; doi:10.1371/journal.pone.0017361)
Supplement: Table S2 — net BRET between β2AR-Rluc8 and V-kras ( Figure 4 ). (DOC) [file pone.0017361.s003.doc]

Table S2: net BRET between β_2_AR-Rluc8 and V-kras (Figure 4).

|  |  | **vehicle** | | | **treatment** | | | **vehicle vs. treatment** | |
| --- | --- | --- | --- | --- | --- | --- | --- | --- | --- |
| **treatment:** | ***n*** | **control**  **net BRET** | **isoproterenol**  **net BRET** | **% change** | **control**  **net BRET** | **isoproterenol**  **net BRET** | **% change** | **controls**  ***P*<0.05†** | **% change**  ***P*‡** |
| **sucrose** | 5 | 0.517 ± 0.003 | 0.332 ± 0.004 | -36 ± 1 | 0.547 ± 0.004 | 0.510 ± 0.005 | -7 ± 1 | yes | <0.001 |
| **4°C** | 5 | 0.479 ± 0.005 | 0.304 ± 0.004 | -37 ± 1 | 0.490 ± 0.005 | 0.456 ± 0.004 | -7 ± 2 | no | <0.001 |
| **arr2 (319-418)** | 6 | 0.414 ± 0.022 | 0.222 ± 0.018 | -47 ± 1 | 0.319 ± 0.020 | 0.185 ± 0.015 | -42 ± 1 | yes | 0.004 |
| **dyn K44A** | 5 | 0.269 ± 0.062 | 0.149 ± 0.047 | -48 ± 4 | 0.259 ± 0.062 | 0.213 ± 0.054 | -18 ± 1 | no | <0.001 |
| **dynasore** | 4 | 0.347 ± 0.026 | 0.188 ± 0.021 | -46 ± 2 | 0.337 ± 0.025 | 0.242 ± 0.019 | -28 ± 2 | no | 0.006 |

†- vehicle control versus treated control, repeated measures ANOVA, Tukey’s multiple comparison.

‡- vehicle % change in net BRET versus treated % change in net BRET, paired t-test.
