# Supplementary material for: Internalization Dissociates β2-Adrenergic Receptors
Source: PLoS One. 2011 Feb 22;6(2):e17361. doi: 10.1371/journal.pone.0017361 (PMC3043075; doi:10.1371/journal.pone.0017361)
Supplement: Table S3 — net BRET between β2AR-Rluc8 wild-type (wt) and binding-defective (DS) mutants and V-kras ( Figure 2A, C ). (DOC) [file pone.0017361.s004.doc]

Table S3: net BRET between β_2_AR-Rluc8 wild-type (wt) and binding-defective (DS) mutants and V-kras (Figure 2A, C).

|  |  |  | **V-kras** | | | |
| --- | --- | --- | --- | --- | --- | --- |
| **donor:** | **other receptor:** | ***n*** | **control net BRET** | **isoproterenol net BRET** | **% change** | ***P*†** |
| **β2AR-Rluc8** | **β2AR D113S** | 4 | 0.227 ± 0.021 | 0.135 ± 0.012 | -40 ± 5 | 0.009 |
| **β2AR-Rluc8** | **β2AR wt** | 4 | 0.223 ± 0.010 | 0.167 ± 0.011 | -25 ± 3 | 0.005 |
| **β2AR D113S-Rluc8** | **β2AR D113S** | 4 | 0.282 ± 0.024 | 0.294 ± 0.024 | 5 ± 3 | 0.190 |
| **β2AR D113S-Rluc8** | **β2AR wt** | 4 | 0.239 ± 0.019 | 0.225 ± 0.017 | -6 ± 1 | 0.022 |
| **β2AR-Rluc8** | **Flag-β2AR D113S** | 4 | 0.171 ± 0.005 | 0.090 ± 0.002 | -47 ± 1 | <0.001 |
| **β2AR-Rluc8** | **Flag-β2AR wt** | 5 | 0.143 ± 0.010 | 0.103 ± 0.008 | -28 ± 2 | <0.001 |
| **β2AR D113S-Rluc8** | **Flag-β2AR D113S** | 4 | 0.198 ± 0.004 | 0.200 ± 0.002 | 1 ± 1 | 0.567 |
| **β2AR D113S-Rluc8** | **Flag-β2AR wt** | 5 | 0.160 ± 0.009 | 0.153 ± 0.008 | -4 ± 1 | 0.022 |

†- control net BRET versus isoproterenol net BRET, paired t-test.
