# Supplementary material for: Internalization Dissociates β2-Adrenergic Receptors
Source: PLoS One. 2011 Feb 22;6(2):e17361. doi: 10.1371/journal.pone.0017361 (PMC3043075; doi:10.1371/journal.pone.0017361)
Supplement: Table S4 — net BRET between β2AR-Rluc8 and β2AR-V wild-type (wt) and binding-defective (DS) mutants ( Figure 4 and Figure S1). (DOC) [file pone.0017361.s005.doc]

Table S4: net BRET between β_2_AR-Rluc8 and β2AR-V wild-type (wt) and binding-defective (DS) mutants (Figure 4 and Figure S1).

|  |  |  | **vehicle** | | | **treatment** | | | **vehicle vs. treatment** | |
| --- | --- | --- | --- | --- | --- | --- | --- | --- | --- | --- |
| **receptors**  **Rluc8/V:** | **treatment:** | ***n*** | **control**  **net BRET** | **isoproterenol**  **net BRET** | **% change** | **control**  **net BRET** | **isoproterenol**  **net BRET** | **% change** | **controls**  ***P*<0.05†** | **% change**  ***P*‡** |
| **wt/wt** | **sucrose** | 6 | 0.201 ± 0.010 | 0.211 ± 0.211 | 5 ± 2 | 0.202 ± 0.008 | 0.194 ± 0.007 | -4 ± 2 | no | 0.026 |
| **wt/wt** | **4°C** | 6 | 0.207 ± 0.004 | 0.222 ± 0.222 | 7 ± 1 | 0.194 ± 0.004 | 0.182 ± 0.011 | -6 ± 4 | no | 0.030 |
| **wt/wt** | **arr2 (319-418)** | 6 | 0.315 ± 0.013 | 0.340 ± 0.340 | 8 ± 2 | 0.218 ± 0.007 | 0.236 ± 0.005 | 8 ± 1 | yes | 0.869 |
| **wt/wt** | **dyn K44A** | 5 | 0.142 ± 0.038 | 0.165 ± 0.165 | 19 ± 3 | 0.125 ± 0.038 | 0.125 ± 0.040 | 0 ± 3 | yes | 0.012 |
| **wt/wt** | **dynasore** | 4 | 0.103 ± 0.010 | 0.113 ± 0.113 | 10 ± 4 | 0.104 ± 0.010 | 0.121 ± 0.010 | 17 ± 2 | no | 0.151 |
| **wt/DS** | **sucrose** | 5 | 0.196 ± 0.001 | 0.162 ± 0.004 | -17 ± 2 | 0.213 ± 0.005 | 0.204 ± 0.005 | -4 ± 1 | no | 0.004 |
| **wt/DS** | **4°C** | 6 | 0.145 ± 0.003 | 0.125 ± 0.003 | -14 ± 1 | 0.150 ± 0.005 | 0.147 ± 0.004 | -2 ± 1 | no | <0.001 |
| **wt/DS** | **arr2 (319-418)** | 6 | 0.258 ± 0.004 | 0.228 ± 0.003 | -12 ± 1 | 0.199 ± 0.004 | 0.179 ± 0.003 | -10 ± 1 | yes | 0.020 |
| **wt/DS** | **dyn K44A** | 5 | 0.143 ± 0.028 | 0.119 ± 0.027 | -18 ± 3 | 0.123 ± 0.022 | 0.116 ± 0.020 | -6 ± 1 | yes | 0.021 |
| **wt/DS** | **dynasore** | 4 | 0.119 ± 0.010 | 0.083 ± 0.009 | -30 ± 2 | 0.120 ± 0.012 | 0.103 ± 0.009 | -14 ± 1 | no | 0.009 |
| **DS/wt** | **sucrose** | 6 | 0.222 ± 0.012 | 0.202 ± 0.013 | -9 ± 3 | 0.227 ± 0.014 | 0.218 ± 0.012 | -4 ± 2 | no | 0.080 |
| **DS/wt** | **4°C** | 3 | 0.280 ± 0.001 | 0.263 ± 0.001 | -6 ± 0 | 0.260 ± 0.005 | 0.259 ± 0.001 | 0 ± 2 | yes | 0.081 |
| **DS/wt** | **arr2 (319-418)** | 7 | 0.315 ± 0.006 | 0.301 ± 0.005 | -5 ± 1 | 0.231 ± 0.007 | 0.218 ± 0.006 | -5 ± 1 | yes | 0.473 |
| **DS/wt** | **dyn K44A** | 5 | 0.147 ± 0.036 | 0.131 ± 0.035 | -12 ± 2 | 0.126 ± 0.033 | 0.123 ± 0.033 | -3 ± 1 | yes | 0.004 |
| **DS/wt** | **dynasore** | 4 | 0.109 ± 0.012 | 0.088 ± 0.011 | -20 ± 2 | 0.113 ± 0.011 | 0.104 ± 0.011 | -9 ± 2 | no | 0.020 |
| **DS/DS** | **sucrose** | 5 | 0.237 ± 0.004 | 0.237 ± 0.003 | 0 ± 1 | 0.250 ± 0.007 | 0.253 ± 0.007 | 1 ± 1 | yes | 0.476 |
| **DS/DS** | **4°C** | 6 | 0.149 ± 0.020 | 0.155 ± 0.021 | 4 ± 0 | 0.145 ± 0.021 | 0.152 ± 0.021 | 5 ± 1 | no | 0.606 |
| **DS/DS** | **arr2 (319-418)** | 6 | 0.288 ± 0.010 | 0.293 ± 0.009 | 2 ± 1 | 0.272 ± 0.006 | 0.274 ± 0.007 | 1 ± 1 | yes | 0.514 |
| **DS/DS** | **dyn K44A** | 5 | 0.157 ± 0.029 | 0.158 ± 0.029 | 0 ± 1 | 0.136 ± 0.025 | 0.140 ± 0.024 | 4 ± 2 | yes | 0.217 |
| **DS/DS** | **dynasore** | 3 | 0.140 ± 0.014 | 0.141 ± 0.016 | 0 ± 1 | 0.143 ± 0.013 | 0.157 ± 0.010 | 10 ± 3 | no | 0.161 |

†- vehicle control versus treated control, repeated measures ANOVA, Tukey’s multiple comparison.

‡- vehicle % change in net BRET versus treated % change in net BRET, paired t-test.
